# Short-Text Classification Detector: A Bert-Based Mental Approach

**DOI:** 10.1155/2022/8660828

**Published:** 2022-03-10

**Authors:** Yongjun Hu, Jia Ding, Zixin Dou, Huiyou Chang

**Affiliations:** ^1^School of Management, Guangzhou University, Guangzhou 510006, China; ^2^School of Data and Computer Science, Sun Yat-sen University, Guangzhou 510006, China; ^3^Research Center of e-commerce, Guangzhou University, Guangzhou 510006, China

## Abstract

With the continuous development of the Internet, social media based on short text has become popular. However, the sparsity and shortness of essays will restrict the accuracy of text classification. Therefore, based on the Bert model, we capture the mental feature of reviewers and apply them for short text classification to improve its classification accuracy. Specifically, we construct a model text at the language level and fine tune the model to better embed mental features. To verify the accuracy of this method, we compare a variety of machine learning methods, such as support vector machine, convolution neural networks, and recurrent neural networks. The results show the following: (1) Through feature comparison, it is found that mental features can significantly improve the accuracy of short text classification. (2) Combining mental features and text as input vectors can provide more classification accuracy than separating them as two independent vectors. (3) Through model comparison, it can be found that Bert model can integrate mental features and short text. Bert can better capture mental features to improve the accuracy of classification results. This will help to promote the development of short text classification.

## 1. Introduction

With the proliferation of online text information, text classification plays a vital role in obtaining information resources [1]. As an efficient and well-known natural language processing technology, text classification can identify the content of a given document and find the relationship between document features and document categories. It is widely used in various fields, such as event detection [2,3], media analysis [4, 5], viewpoint mining [6, 7], and predicting product revenue [8,9]. Although text classification has always been a well-known problem, a suitable solution for short text classification has not been found. Especially, with the rapid growth of the digital media scale, a complex environment will affect the results of text content retrieval and analysis. This makes short text classification a challenging task. Therefore, to promote content analysis of online text information, a reliable text classification tool is needed [10].

Recently, a large number of scholars have studied text classification. Traditional classification algorithm models include *K*-nearest neighbor (KNN) [11], naive Bayes (NB) [12], and support vector machine (SVM) [13]. These models have good classification results and have been widely used. They extract the features of text documents and then use one or more classifiers to predict multiple related tags [14, 15]. However, such methods are time-consuming and require the extensive domain knowledge of experts [10]. At present, with the development of deep learning, the traditional classification methods are gradually integrated and replaced by deep learning classification algorithms. As deep learning can learn representation from data without complex feature engineering, it has become a hot research topic in this field [16, 17]. To obtain a better classification effect, many researchers use a convolutional neural network (CNN) [18] and recurrent neural network (RNN) [19] to extract and calculate text features. In particular, the bidirectional encoder representations from transformers (Bert) [20] developed by Google. Different from the previous network architecture, Bert is based on attention mechanism and transformer coding structure. However, previous studies have not used the mental feature of the speaker, that is, mental features, for short text classification.

The current research challenges are as follows. Although the emergence of machine learning will improve the effect of text classification, the sparsity and shortness of text will limit the accuracy of text classification. At the same time, with the growth of smart phones, short text has been integrated into daily life. Therefore, a method is needed to quickly identify the publisher's intention, to improve the accuracy of classification. In addition, when it comes to cross languages, the traditional method uses the same corpus to train the classifier, but this method cannot be extended to multilingual environment [21]. To address this knowledge gap, in this research work, we focus on text classification according to users' mental features, and use cross-lingual data sets for experiments. Given this, this paper proposes a method, which effectively integrates mental features with text content on a linguistic level. Specially, we have designed two methods to integrate text context and all features on a linguistic level. Then, we fine-tune the model by evaluating the significance of each feature. The mental features can reflect the speaker's behavior and improve the accuracy of the method. Meanwhile, to verify the accuracy of this method, we compare a variety of machine-learning methods. The results show that our method has significant advantages in short text classification tasks.

The main contributions of this paper are as follows:

–A Bert-based mental approach is proposed for the classification of short text content. The proposed method combines the user's mental feature with the short text content. It can help better identify the user's intention contained in the short text, that is, false comments or text topic detection.

–Compared with other existing machine-learning research, the proposed method effectively integrates mental feature vector and short text vector. It improves the accuracy of text classification and achieves good accuracy on cross-corpus data sets.

The rest of this paper is shown as follows.  Section 2 summarizes the literature reviews. In Section 3, we introduce the methodology.  Section 4 is an experiment, including data set, research settings, evaluation metrics, and experimental results.  Section 5 discusses the key findings, theoretical implications, and practical implications. Finally, the conclusion is presented.

## 2. Literature Review

### 2.1. Mental Theory

The concept of mental theory was first proposed by Craik [22]. The basic assumption of the mental theory is that any form of communication is based on the situational way people talk about [23, 24]. It represents how people imagine and understand the situation in the world. Although it may contain factual information, it is not only able to identify facts, it can also be used to make judgments and inferences, to affect people's behavior. When people use signs to represent objects, it is often quite abstract. Therefore, mental models need to be used to explain existing concepts by adding more information [25], which will have a potential impact on people. Mental theory has been widely used in many fields, such as management education [26] and management decision-making [27, 28].

In the field of text, the mental theory has been proved to improve text comprehension [29–31]. Specifically, mental theory reflects the different levels of representation formed by the speaker in the process of text writing. Representation refers to an abstract propositional representation between the thought contained in the text and its linguistic information. This is also a cognitive representation of reality, which is related to the speaker's cognition, perception, and behavior in various situations [25, 32]. It is worth noting that it represents the content of the text (the events, objects, and processes described in the text), rather than the characteristics of the text itself [33].

At the same time, deep learning can be used as a powerful tool to expand mental theory. For example, to improve teaching efficiency, Tawatsuji et al. [34] and Matsui et al. [35] extracted the relationship between students' mental state and mental information through deep learning, supplemented by teachers' speech behavior. To ensure driving safety, Darwish et al. [36] analyzed the driver's psychology through in-depth learning and judge how the driver perceives the environment. Dutta et al. [37] believed that machine-learning algorithm is used as a classification tool of mental state, which can improve the accuracy of classification.

Therefore, this paper combines mental theory with deep learning to apply the classification of short texts. By discovering the relationship between text and mental features, more accurate classification results will be obtained.

### 2.2. Text Classification Method

Recently, most classification methods are mainly based on machine learning. In terms of KNN, Moldagulova et al. [11] and Trstenjak et al. [38] use the KNN algorithm to classify documents. The results show that this algorithm has good classification performance. In terms of Xgboost, Wang et al. [39] took the Xgboost algorithm and granularity parameters as input characteristics to predict sample categories. Li and Zhang [40] proposed a classification prediction model based on the Xgboost algorithm. In terms of NB, Zhu et al. [12] used the NB algorithm for text classification. Jiang et al. [41] proposed an improved NB technology for text classification performance. This method solves the problem of unsatisfactory results caused by the uneven distribution of training data. Bilal et al. [42] used the NB algorithm for periodical literature classification. The results show that the accuracy of this method is high enough. In addition, some scholars also studied SVM, which is a method used to predict and define how to classify data sets [43]. It can classify text data into predefined classes [44]. In terms of SVM, Luo [13] applied the SVM model to the classification of English texts and documents. The results show that this method has good performance. According to Vijayarani et al. [45], the accuracy of SVM is slightly higher than that of NB. However, traditional target classification focuses on feature engineering to maximize the use of classifiers [46–48], such as SVM. Such methods are time-consuming and require the extensive domain knowledge of experts.

With the development of machine learning, a large number of NN models have emerged in natural language processing tasks [49,50]. As these methods can learn representation from data without complex feature engineering, NN has become a hot research topic in this field. Mainstream NN include RNN [19,51], gated neural network (GNN) [52,53], CNN [50, 54, 55], and long short-term memory (LSTM) [56]. The most popular neural network architectures are CNN and RNN. CNN has a good performance on features extraction by convolution kernel, which improves the accuracy of feature descriptors. RNN is widely used to capture flexible context information. However, Kandhro et al. [57] found that the performance of the LSTM method has more advantages than CNN and RNN. LSTM solves the vanishing gradient problem and has long-term dependence, which can retain the characteristics of previous learning [56]. Tang et al. [58] found that LSTM can effectively capture the information of sentences. Lee et al. [59] mine tourists' destinations and preferences through text classification and spatial clustering based on LSTM. The results show that this method has good results. Subsequently, Google proposed Bert [20]. This model has made a breakthrough in the text field and achieved the most advanced results. A large number of scholars widely used Bert, which is enough to prove its great advantages in feature extraction [60,61].

However, previous studies have not used human mental-related features for short text classification. We assume that in a specific linguistic pattern, mental features can provide more information for short text classification. Therefore, this paper studies the Bert method for short text classification, and fine-tune it. Specifically, to obtain more accurate classification results, we combine mental features with text content according to specific short text patterns.

## 3. Methodology

### 3.1. Theory

**Figure 1:**
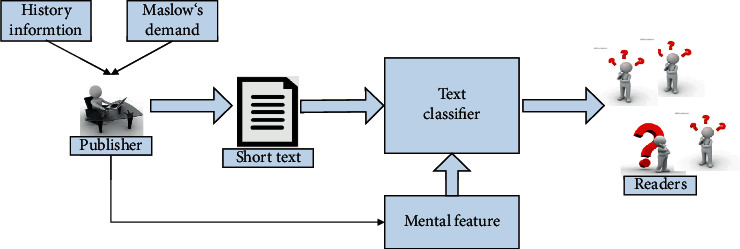
The concept of text classification based on mental feature.

The sparsity and shortness of short text may seriously destroy the representation of short text. An important solution is to enrich the short text representation by involving the cognitive aspects of the text, such as mental features. Generally, the short text content of users can be enriched from external mental and internal mental features (as shown in Figure 1).

Figure 1 shows the conceptual framework proposed in this study, which is divided into two main stages: mental model processing and classification model training. First, we propose two methods to embody mental features, namely, history information and Maslow's need. Our text data set contains cross-lingual corpus. The approach proposed in this paper can effectively integrate mental feature vector and short text vector, classify short text with higher accuracy, and help readers understand the intention contained in the text. Next, the application of nonconcentric zhite is introduced in methods 1 and 2, respectively.

#### 3.1.1. Method 1

**Figure 2:**
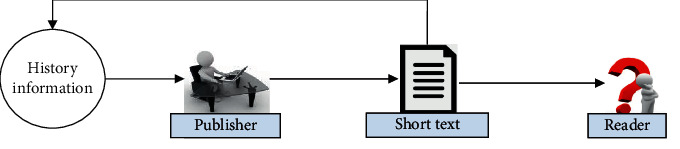
The concept of text classification based on history information.

The historical information of users contain their behavior laws. There will be great differences in the historical information of different users. Based on this, this paper introduces the user history feature into the short text classification model as an external mental feature. Its concept is as shown in Figure 2. By enriching the user's intention expressed in a short text, accurate classification is carried out.

#### 3.1.2. Method 2

**Figure 3:**
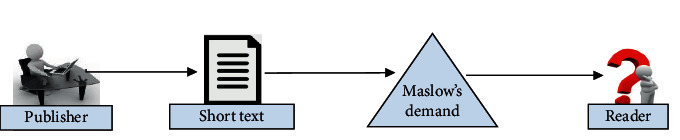
The concept of text classification based on Maslow's need.

This paper takes Maslow's need as the internal mental feature. Maslow's needs include five levels of cognitive needs, including physiological needs, security needs, emotional needs, respect needs, and self-realization needs. It can be expected that different levels of needs play an important role in everyone's character formation. Therefore, it is also important to understand these basic requirements in short text classification. If the mental feature is constructed according to the different needs of users, the short text can be enriched, and the readers can better understand the content of the text. Its concept is as shown in Figure 3.

### 3.2. Model Structure

**Figure 4:**
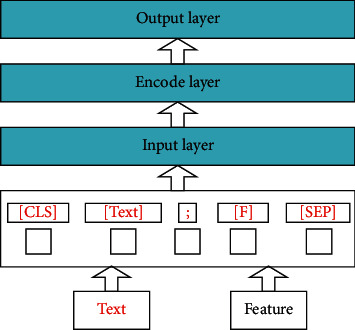
Basic structure of the Bert.

The Bert is realized by constructing mental features and combining domain-related knowledge. Its structure is shown in Figure 4. The model integrates text and features into a corpus instead of inputting them with different vector matrices. The data input rules can be described as follows:

- [Text] indicates the text content
- [F] represents a Feature item
- [CLS] indicates that the corpus is used for the classification model
- [SEP] represents a clause symbol, which is used to disconnect two sentences in the input corpus

The Bert mainly consists of the following three parts:

  Input layer: the feature and text data are used to establish the input sequence for this model. Then, the final input representation is obtained by summing the position embedding, word embedding, and segmentation embedding of each tag sequence.
  Encoder layer: it consists of 12 transform blocks, which input the marked sequence and output the representation of the sequence.
  Output layer: it consists of a simple softmax classifier at the top of the encoder layer, which is used to calculate the conditional probability distribution on the predefined classification label.

#### 3.2.1. Input Layer

The difference between the two methods is how we input meta-data into the Bert:

- Pair Method (PM) : this method inputs the feature text into the model as a sentence independent of the claim. That is, at the token level, the claim is separated from the feature text by the special token '[SEP]'.
- News Text Method (NTM) : this method inputs the news text into the model as a single sentence. Noteworthy, the News Text is composed of Claim and Feature Text. They are separated by '; '. Only one '[SEP]' token is added at the end of the entire token sequence.

**Figure 5:**
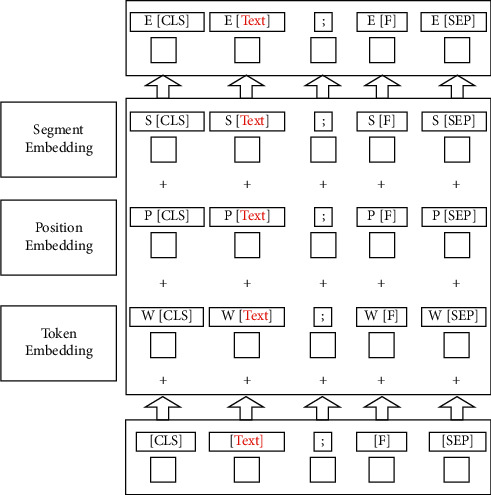
Construction of input sequence representations for Bert.

The input representation of each token *e* is obtained by adding its token embedding (*W*), segment embedding (*S*), and position embedding (*P*). For visualization of this structure, see Figure 5. The embedding features of these three words are as follows:

1. Token embedding: this embedding is a vector to convert each word into a fixed dimension. The input text will be tokenized when it is sent into this embedding. In addition, two special tokens, [CLS] and [SEP], will be inserted at the beginning and the end of the tokenization result. They are regarded as the following classification tasks and the effect of dividing sentences on services, respectively.
2. Position embedding: this embedding refers to encoding the position information of words into feature vectors. It is a crucial link to introduce the word position relationship into the model.
3. Segment embedding: there are only two vector representations of this embedding. The former vector assigns 0 to each token in the first sentence, and the latter vector assigns 1 to each token in the second sentence. If the input has only one sentence, its segment embedding is all 0.

#### 3.2.2. Encoder Layer

**Figure 6:**
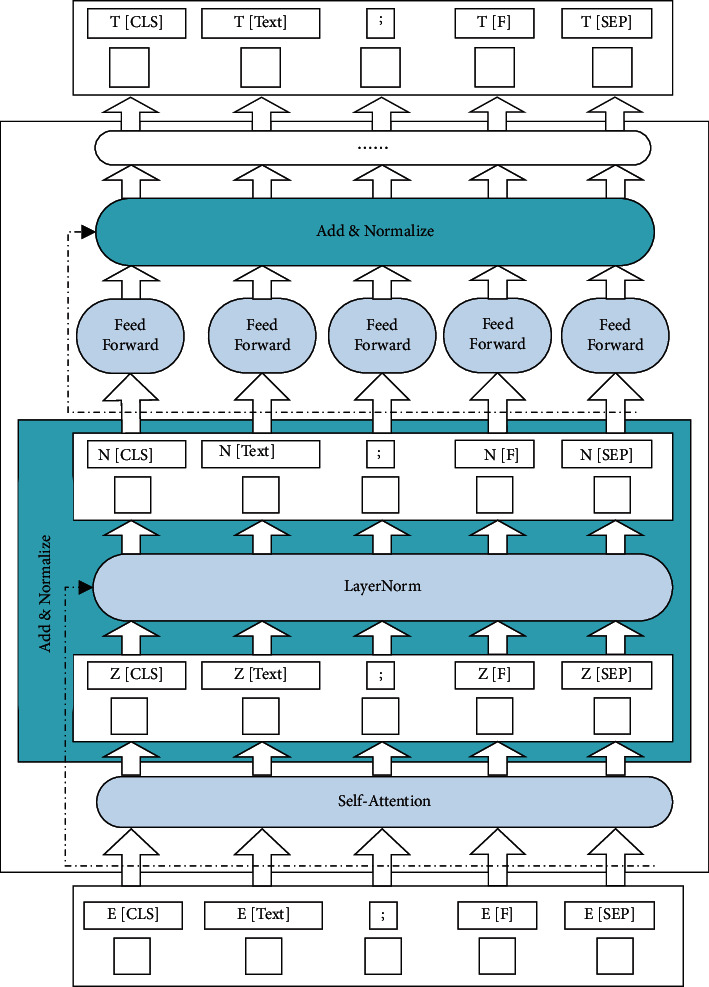
The basic structure of the encoder.

The model architecture is composed of 12 layers of transformers. Its basic structure is shown in Figure 6. Each transformer is composed of a self-attention module, add&norm module, feed-forward module, and add&norm module:

(1) Self-attention module: this module is to find the correlation between words. Each self-attention mechanism first converts the input data into three vectors through three parameter matrices *Q*, *K*, and *V*. Where, *Q* is the query vector parameter matrix, *K* is the key vector parameter matrix, and *V* is the value vector parameter matrix. The converted vector dimension is smaller than the input dimension. Then, the machine calculates the self-attention vector of the *Q*. The main process is to divide the *Q* and the *K* by the square root of the *K*. This will reduce the vector size to a certain extent, which is conducive to keeping the gradient stable during backpropagation. After that a softmax operation is performed on all normalized dot products. Its purpose is to normalize, which can strengthen the influence of relevant time step data and weaken the influence of irrelevant time steps. Finally, multiply the aforementioned results by *V*. The calculation of self-attention is shown in the following formula:
  (1) $$\begin{array}{c} Z=\text{Attention}\left(Q,K,V\right)\\ ={\text{softma}}_{\text{.}}\text{x}\left(\frac{QK^{T}}{\sqrt{d_{t}}}\right)V, \end{array}$$
where *Z* is the output vector of the attention module.
(2) add&norm module: in this module, the *Z* vector is input into LayerNorm, which normalizes *Z*. The purpose of this is not to let the *Z* vector to fall in the saturation region of the activation function. Therefore, the normalized *N* vector is obtained.
(3) Feed-forward module: as the calculation in the self-attention module is linear, to improve the nonlinear fitting ability of the model, a feed-forward network needs to be connected behind it. The network consists of two linear mapping parts. The first part is linear mapping and nonlinear activation relu function. The second part is a linear mapping. The formula is as follows:
  (2) $$\begin{array}{c} F\left(Z\right)=\text{relu}\left(0,ZW_{1}+b_{1}\right)W_{2}+b_{2}, \end{array}$$

where *F* is the output vector of the feed-forward neural network, *W*_1_ and *W*_2_ are the weight matrix, *b*_1_ and *b*_2_ are the bias.

Then the output of the feed-forward network is normalized by the add&norm module.

The aforementioned steps are a transformer. After 12 times, the output of the 12th transformer is a hidden state vector, that is, the *T* vector.

#### 3.2.3. Output Layer

The output layer is a simple softmax classifier at the top of the model. This model only uses the final hidden state vector *T*[CLS] as the aggregate representation of the sequence, that is, the *T* vector output through the transformer. The final classification result is obtained according to the following formula:

(3) $$\begin{array}{c} P={\text{softma}}_{\text{.}}\text{x}\left(T\left[CLS\right]V^{T}\right)=\frac{\exp\left(P\left(y_{i}|T\left[\text{CLS}\right],\theta \right)\right)}{\sum_{i=1}^{c}\exp\left(P\left(y_{i}|T\left[\text{CLS}\right],\theta \right)\right)}, \end{array}$$

where *V* ∈ *R*^*c*^*∗*^*h*^ is the trainable task-specific parameter matrix, and *c* is the number of labels. *h* is the dimension with a default value. It is worth noting that the category *y*_*i*_ with the probability of occurrence is the category to which the *T*[CLS]. Therefore, the final distribution function will output a *C*-dimensional vector. Each dimension represents the probability that *T*[CLS] belongs to *y*_*i*_. When the *C*-dimensional vector elements are normalized, the sum of them is 1.

## 4. Experiment

### 4.1. Data Set

#### 4.1.1. For English Fake News Detection

The data set we experimented with is based on the LIAR data set, which was published by Wang [62]. It consists of a large number of claims, namely text content and related features. These features include subject, speaker, job, state, party, history, and context. Where Claim is the text content (Text). History indicates the speaker's statistics on the historical behavior of news speakers lying. From the perspective of psychology, this behavior can fully describe an individual's psychology. Therefore, this paper takes this index as a mental feature. For truthfulness, it is labeled as true, mostly true, half-true, barely-true, false, and pants-on-fire by journalists. To avoid these insignificant symbols affecting the results, we replaced some specific punctuation marks.

#### 4.1.2. For Chinese Topic Detection

The data set we experimented with is based on social messaging data. The data set is a calculation of a great amount of text content and the Maslow's need features. We regard this feature as a mental feature because it can effectively reflect the mental state of the text publisher. For topic, it is labeled as meaningless, work/study, family, affection, leisure, and a blessing. To avoid these insignificant symbols affecting the results, we replaced some specific punctuation marks.

### 4.2. Experiment Settings

To explore the effectiveness of text classification, we first fine-tune the Bert method. Then, we evaluate the three methods, including all the features. Moreover, we use different feature combinations to evaluate the significance of mental features. Specifically, for model training, we set the learning rate to 2e–5, the batch size to 8, and the training time to 3.0.

### 4.3. Evaluation Metric

For evaluation metric, we use the following equation:

(4) $$\begin{array}{c} \text{accuracy}=\frac{\left(\text{TP}+\text{TN}\right)}{\left(P+N\right)}, \end{array}$$

where TP is for true positive, *P* is for total positive, FN is for false negative, and *N* is for total negative. By calculating these values, we can get the accuracy of the results.

### 4.4. Experimental Results

#### 4.4.1. Fine-Tuning Analysis for Bert

We first compare the two input methods with all features included of Bert, namely PM and NTM. From Table 1, the accuracy of NTM results is 0.476 in English Fake News Detection (EFND) and 0.960 in Chinese Topic Detection (CTD), respectively. Both of them are significantly better than PM. The main reason is that PM inputs text and features as separate sequences. Although the model can learn the representation of each sentence sequence through fine-tuning, due to the segmentation of text and features, it will not be able to associate any feature information with the text, effectively. In other words, as text and features are input as different sentences, some information may be lost, which is the main reason for poor performance. We only need to input the text and features as a whole sentence. It is not necessary to split it with [SEP].

#### 4.4.2. Feature Selection and Analysis

To verify that mental features contribute most to short text classification, we experimented with NTM composed of text and single feature items in the Bert method.

From Table 2, it can see that the model performs worst when it is fine-tuned only with text in EFND. Meanwhile, different features make different contributions to fake news detection. The mental feature has the greatest improvement, and their accuracy is more than 0.4 in EFND. This shows that mental features can effectively improve the accuracy of fake news detection.

In Table 3, we also found that the accuracy of text content is much lower than that of mental features in CTD. It verifies the contribution of mental features to topic detection.

#### 4.4.3. Comparative Text Classification Method

In this section, we first compare our method with existing methods, using text and mental feature data. From Tables 4 and 5, the performance of Bert is significantly better than other models. It improves the accuracy by 0.2. This confirms the effectiveness of Bert. It also shows that the self-attention mechanism has a better ability to capture sequence semantics. Based on ensuring the task awareness of the model, it can directly learn the relationship between the target text sequence and the corresponding classification label, which simplifies the training.

In addition, when only plain text is used, our method is also used to compare existing models. In Tables 4 and 5, whether EFND or CTD, the results show that the performance of models using the mental feature is significantly better than those using plain text data. The mental feature approximately improves the absolute value of accuracy by 0.2.

In summary, the experimental results of EFND and CTD are consistent, indicating that mental features play a key role in short text classification, whether false news detection or topic detection. In especial, our method, regardless of whether it has mental features or not, the result is the best. Therefore, Bert not only has higher accuracy but also has universality. In other words, it can be applied to cross-linguistic and multidomain.

## 5. Discussion

### 5.1. Key Findings

The purpose of this study is to explore the role of mental features in short text classification. In general, our findings show that there are two factors in the mental feature of information publishers. The first kind of factor is an external mental feature, that is, historical information. The data show that the classification result of the model is more accurate under the joint action of short text and historical information. This shows that the authenticity of the short text of the information published is affected by its subject. For those who publish false reviews more often in history, the probability of new short texts being false will increase. By combining the effective features extracted from short text, we can better understand the authenticity of publishers.

The second type of factor is the internal mental feature, that is, Maslow's need. The results show that this feature is related to the accuracy of short text classification. This feature reflects the publisher's deep-seated aspects, that is, psychological changes. This shows that the content of short texts can be enriched by depicting the psychological changes of information publishers. People will have different psychological changes when publishing different texts. By taking psychological changes as auxiliary features, they can map with text features. It can improve the accuracy of text topic classification.

In summary, short text cannot completely reflect the meaning that users want to express. Through mental features, users' intentions expressed in short texts can be enriched.

### 5.2. Theoretical Implications

We apply mental features to short text classification. Specifically, we prove through experiments that the improvement of short text accuracy is achieved through the integration of mental and text features. In addition, through experiments, we find the form in which these two features are input into Bert to achieve more effective fusion.

Our results are better than traditional methods. To improve the accuracy of classification results, learning mental features through the Bert model and recognizing their relationship with short texts. This provides an innovative perspective and enriches the literature of short text classification.

### 5.3. Practical Implications

False review identification is very important. With the vigorous development of the Internet economy, the credibility of reviews is of great significance to consumers. False reviews imitate the tone of real reviews, which makes it difficult to distinguish between true and false. Its content distorts the facts and misleads consumers, which has a great negative impact on the interests of consumers and the platform. From an academic point of view, there is still a huge research space for the exploration of this direction, which is very worthy of in-depth excavation by interested researchers. By mining more consumers' mental features and depicting users' mental portraits, the users' credibility can obtain. Combining it with the text content, it is expected to effectively identify the authenticity of reviews.

Text topic classification method is the key technical basis of network public opinion analysis. Internet public opinion refers to people's opinions or remarks with certain influence and tendency on the Internet with the help of Internet media. With the rapid growth of communication technology and intelligent devices, there has been a huge surge in data traffic. Different applications, users, and devices generate large amounts of data every second [63]. Once the wrong or extreme public opinion is spread, with its influence in the network world, it will often cause huge public opinion pressure and even uncontrollable consequences. Therefore, it is necessary to control the dynamics of public opinion. Through the in-depth mining of users' mental features, the performance features of network public opinion can be reflected. It is hoped that it can provide a reference for effectively understanding the evolutionary process of network public opinion.

## 6. Conclusion

Due to the sparsity of the short text, it is difficult for the machine to understand its content. We find two mental feature that reflect the information publisher. By integrating this feature with the text, the accuracy of short text topic classification can be improved. In this context, we propose a method which effectively integrates mental features with text content on a linguistic level. Specially, we have designed two input methods to integrate text context and feature on a linguistic level. Namely, the pair method and new text method. Also, to verify the accuracy of Bert method, we compare a variety of machine-learning methods, such as SVM, CNN, and RNN. The results show that (1) through feature comparison, it is found that mental features can significantly improve the accuracy of short text classification. (2) Combining mental features and text as input vectors can provide more classification accuracy than separating them as two independent vectors. Namely, the new text method is better than the pair method. (3) Through model comparison, it can be found that the Bert model can integrate mental features and short text. Bert can better capture mental features to improve the accuracy of classification results.

There are still some limitations in this paper. Our results demonstrate the effectiveness of mental features in false review detection and topic classification. However, in different situations, the interference factors are different. Therefore, it will be interesting to test our method for this study in other contexts. We encourage other researchers to take mental features as a meaningful framework and integrate different features to improve the accuracy of short text classification. In addition, this paper classifies short text on offline data sets. Therefore, in the future, we expect to automatically record users' daily information data to facilitate real-time analysis of users' mental status. In this way, short text can be detected in real-time.

## Figures and Tables

**Table 1:** The result of Bert in different methods.

	EFND	CTD
PM	0.265	0.754
NTM	0.460	0.960

**Table 2:** The results with different features in EFND.

EFND	Accuracy	EFND	Accuracy
Only text	0.273	Text + State	0.293
Text + Subject	0.281	Text + Party	0.288
Text + Speaker	0.284	Text + Context	0.288
Text + Job	0.285	Text + Mental	0.459

**Table 3:** The results with different features in CTD.

CTD	Accuracy	CTD	Accuracy
Text + Mental	0.960	Only text	0.780

**Table 4:** The result of different methods in EFND.

	Accuracy	
Model	Text + Mental	Only text
Bert	0.460	0.273
Bays	0.243	0.236
SVM	0.259	0.256
Xgboost	0.250	0.217
KNN	0.222	0.206
CNN	0.165	0.155
LSTM	0.166	0.155
GRU	0.164	0.1437
BP	0.164	0.159
RNN	0.162	0.158

**Table 5:** The result of different methods in CTD.

	Accuracy	
Model	Text + Mental	Only text
Bert	0.960	0.780
Bays	0.582	0.564
SVM	0.578	0.564
Xgboost	0.577	0.575
KNN	0.564	0.397
CNN	0.550	0.497
LSTM	0.529	0.525
GRU	0.552	0.527
BP	0.533	0.527
RNN	0.512	0.496

## Data Availability

For English fake news detection (EFND), the data set we experimented with is based on the LIAR data set (http://www.cs.ucsb.edu/∼william/data/liar_dataset.zip); For Chinese topic detection (CTD), the data set we experimented with is based on social messaging data. We welcome interested partners to contact the first author for data (hyjsdu96@126.com).
